# Osteomyelitis affecting mandible in tuberculosis patients

**DOI:** 10.4317/jced.50588

**Published:** 2012-02-01

**Authors:** Freny Karjodkar, Vasu S. Saxena, Anuradha Maideo, Subodh Sontakke

**Affiliations:** 1 MDS. Professor and Head, Department of oral Medicine and Radiology, Nair Hospital Dental College, Mumbai; 2 BDS. Resident, Department of Oral Medicine and Radiology, Nair Hospital Dental College, Mumbai; 3 MDS. Assistant Professor, Department of Oral Medicine and Radiology, Nair Hospital Dental College, Mumbai

## Abstract

Tuberculosis (TB) is a frequent health problem in developing nations. It has two forms pulmonary and secondary causing other kinds of TB, collectively denoted extra pulmonary tuberculosis. The prevalence of extra pulmonary TB has increased in the last couple of years. Maxillofacial manifestations of tuberculosis form nearly 10% of all extra pulmonary manifestations of the disease. Extra pulmonary TB involving maxillofacial region is our prime concern. Very few cases of TB of the temporomandibular joint (TMJ) and mandible have been reported in literature. The clinical appearance of TB infection of the TMJ has been described as unspecific, resembling arthritis, osteomyelitis, cancer or any kind of chronic joint diseases. This article describes two cases where the bone, namely TMJ and angle of mandible are affected by tuberculosis. In addition to conventional radiographs we used Cone Beam Computed tomography (CBCT) to explore the third dimension of the lesion.

** Key words:**Tuberculosis, bone, osteomyelitis, CBCT.

## Introduction

The impact of tuberculosis (TB) falls mainly on developing nations. In most industrialized countries, the annual numbers of cases and deaths caused by TB have steadily declined over the past century up to the mid-1980s. Since then, an increasing number of TB cases in immigrants have reversed this downward trend in several countries (1). Tuberculosis of bone is an uncommon form of chronic osteomyelitis, occurring more often in young individuals and usually in late stages of the disease. Flat bones, including those of the skull and mandible, are rarely affected. Due to the rarity of tuberculosis of mandible, it seldom arouses clinical suspicion, especially when a positive history of a systemic infection or therapy is denied (2).We discussed here two illustrative cases of tuberculous osteomyelitis and its manifestation in maxillofacial region. We used Cone Beam Computed tomography (CBCT) to explore the third dimension of the lesion to diagnose the destructive extent of the lesion.

## Case report 1

An 18 year old female patient was reported to the department of Oral Medicine and Radiology in Nair Hospital and Dental College; with complain of swelling on left side of face in the preauricular region. She had a history of swelling since 8 months which was slowly increasing in size. She had no prior medical or family history of treatment for any chronic infective disease. Patient was thin built and moderately nourished. The patient pre-sented with trismus and a moderate sized swelling present on left side of face in the preauricular region with no sinus or discharging pus. Overlying skin was normal in colour and ear lobule was not elevated. Swelling was tender and firm on palpation. A lymph node was palpable, tender and of 1.5 x 1.5 cm size in left submandibular area. Presence of trismus restricted examination of oral cavity; an ulcerative lesion over the retro molar region in relation to impacted 38 was seen. Tenderness was present over the retro molar region in relation to 38. Provisional differential diagnosis came as space infection/ pericoronitis in relation to impacted 38/ periapical abscess.

**Figure 1:**
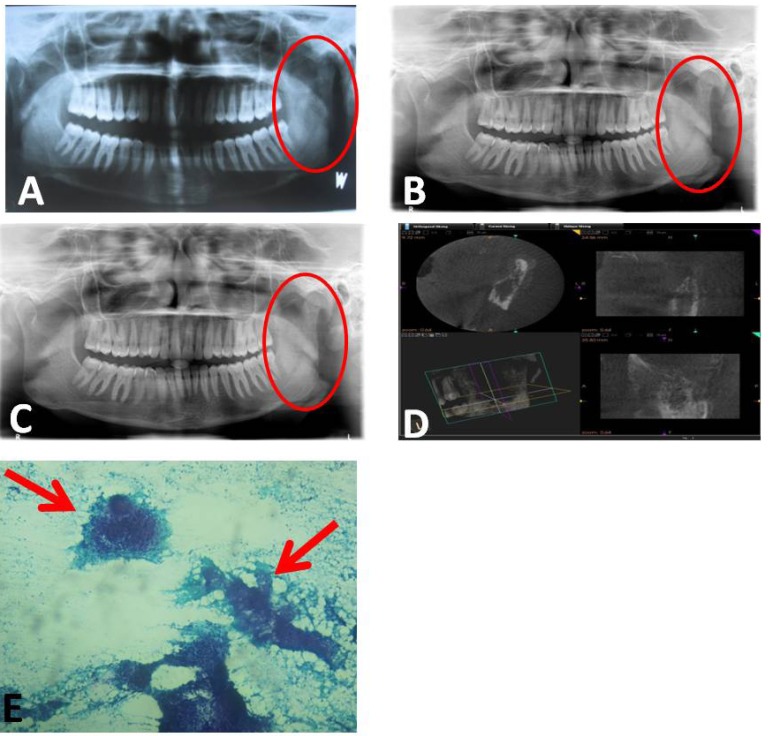
Panoramic View: showed diffuse radiolucency in the ramus of mandible with loss of cortication on the superior and anterior portion of condyle, the extent of destruction observed lead to the suspicion that a larger lesion may be present within the ramus. B. Follow up view after 2 months. C. Follow up view after 5 months. D. Cone Beam Computed Tomograph (CBCT): The left TMJ showed pronounced rarefaction and destruction of bone in the ramus with discontinuity of the cortical boundary suggestive of perforation and erosion of the condyloid head. E. FNAC slide showed purulent material aspirated and smear showed abundant caseous necrosis, occasional epithelial cell granulomas, aggregated polymorphs and few lymphocytes.

In radiographic findings (Fig. 1) panoramic view showed diffuse radiolucency in the ramus of mandible with loss of cortication on the superior and anterior portion of condyle, the extent of destruction observed lead to the suspicion that a larger lesion may be present within the ramus (Fig 1- A). Cone Beam Computed Tomograph (CBCT) of the left TMJ showed pronounced rarefaction and destruction of bone in the ramus with discontinuity of the cortical boundary suggestive of perforation and erosion of the condyloid head (Fig 1- D). Radiographic diagnosis came as Osteomyelitic changes in relation to left condyle and ramus.

Ultrasound finding showed an approximately 4 x 1 cm. sized hypoechoic collection with internal echoes within, is seen in muscular plane with erosion of underlying mandible.

Impression came as Left mandibular ramus osteomyelitis with collection.

Fine needle aspiration cytology (FNAC) (Fig 1-E) of the left parotid region swelling showed purulent material aspirated and smear shows abundant caseous necrosis, occasional epithelial cell granulomas, aggregated poly-morphs and few lymphocytes. Histological findings came as cold abscess, suggestive of tuberculosis. Final diagnosis came as extra pulmonary tuberculosis with osteomyelitis. Patient was referred to medical department where after confirming diagnosis she was started with Directly Observed Therapy, Short Course (DOTs) and was kept on periodic follow up. The patient was examined after 2/5 months of treatment, the extra oral swelling had resolved and panoramic radiographs (Fig 1-B, C) showed progressive bone healing in the ramus and increase in cortication of left condylar head.

## Case Report 2

A 45 year old male patient came to Oral Medicine and Radiology in Nair Hospital Dental College; with complain of swelling on right side at the angle of the jaw since 2 months. Patient had pain in the lower right posterior teeth since 4 months. She had under gone an extraction of the lower right first, second and third molars under antibiotic cover one month ago. Post extraction, the patient gave history of reduction of the swelling, but once the medication was stopped the swelling started gradually increasing in size. Patient had history of cough since 5-6 months and evening rise in temperature since 10 days. Patient was conscious, cooperative, and afebrile. Extra oral examination showed a solitary round swelling (3x4 cm) with diffuse borders in right angle region and temperature of overlying skin was raised. Swelling was tender on palpation but no lymph node was palpable. Intra oral examination showed missing mandibular molars on the right side. Edentulous ridge appeared normal with no swelling. Provisional differential diagnosis came as space infection/ residual infected cyst.

**Figure 2:**
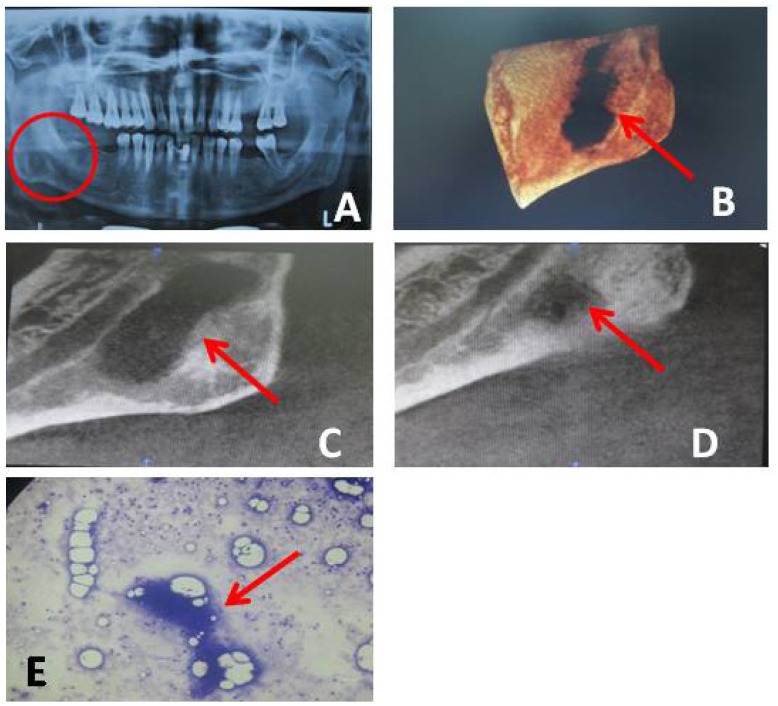
Panoramic view: showed ill defined radiolucency with sclerotic borders located below mandibular canal on right side. Cone Beam Computed Tomography (CBCT): reconstructed view of the lesion showed marked irregular destruction at the angle region. Cone Beam Computed Tomography (CBCT) showed destruction in close proximity and below the inferior alveolar canal near Posteroinferior border of ramus and lower border of body of the mandible on right side. Follow up view showed marked bone formation. D. Cone Beam Computed Tomography (CBCT) showed healing of the lesion after 4 months Of follow up. E. Fine Needle Aspiration Cytology (FNAC) showed necrotic material, degenerated polymorphs, isolated epithelial cells and cluster of epitheloid cell granulomas suggestive of granulomatous inflammation. Acid fast bacilli were not seen in the lymph node.

In radiographic investigations (Fig. 2) panoramic view showed ill defined radiolucency with sclerotic borders located below mandibular canal (Fig 2- A). Cone Beam Computed Tomography (CBCT) showed marked irreg-ular destruction in close proximity and below the inferior alveolar canal near posteroinferior border of ramus and lower border of body of the mandible on right side (Fig 2- B, C). Radiographic diagnosis came as osteomyelitis/ malignancy/ submandibular gland depression. In chest X-Ray no abnormality detected. Ultrasonography (USG) showed ill defined hyperechoic lesion with moving internal echoes, irregular wall in right infra parotid region suggestive of abscess cavity. Montoux test showed positive result with indurated and erythematous area (12mm x 13mm) after 48 hours. Fine Needle Aspiration Cytology (FNAC) of right submandibular lymph node showed necrotic material, degenerated polymorphs, isolated epithelial cells and cluster of epitheloid cell granulomas suggestive of granulomatous inflammation. Acid fast bacilli were not seen in the lymph node (Fig 2-E). Deoxyribonucleic acid-Polymerase Chain reaction (DNA-PCR) showed positive response and mycobacterium tuberculosis. Final diagnosis came as tuberculous osteomyelitis. Patient was started on anti Kochs therapy and kept on follow up. 2 months after starting treatment the patient came with an increased in swelling and extra oral draining sinus. The patient was asked to continue the medication and one month follow up showed reduction in the size of the swelling and healing of the sinus with scar formation. CBCT taken 4 months after starting treatment showed reduction in the bony defect with progressive bone deposition (Fig 2- D).

## Discussion

After a decrease of TB in developed countries up to the mid-1980s, the incidence of TB has been increasing steadily in many countries during the last two decades (3). This is especially related to the increased population of immunocompromised patients and the recent increase of imported cases from immigrants (4). In the USA the TB incidence rate is four times higher in the immigrant population than in native-born citizens (5). In several European countries the proportion of immigrants among persons reported as having TB exceeds 50%. Not only TB of the lungs, but also the extra pulmonary forms, including head and neck TB, has increased disproportionately. The most frequent manifestation of head and neck TB (95%) is cervical lymphadenitis (6). Primary TB of the TMJ is rare. A few cases describe secondary TB infections of the TMJ originating from a fistulous communication from the middle ear (7). Only five cases have been reported of a primary manifestation of TB in the TMJ (4,8). The clinical appearance of TB of the TMJ is non-specific and may be similar to that of arthritis, osteomyelitis or chronic joint diseases. The most common symptom is a painful preauricular swelling (unresponsive to antibiotics), associated with Trismus (9), as seen in our “Case 1”. The involvement of the mandible by tuberculous infection is rare as it contains less cancellous bone (10). Mandible involvement is more frequent than maxilla and the alveolar and angle regions have greater affinity as observed in “Case 2”. Various ways of dissemination are described in the  Table 1.

**Table 1:**
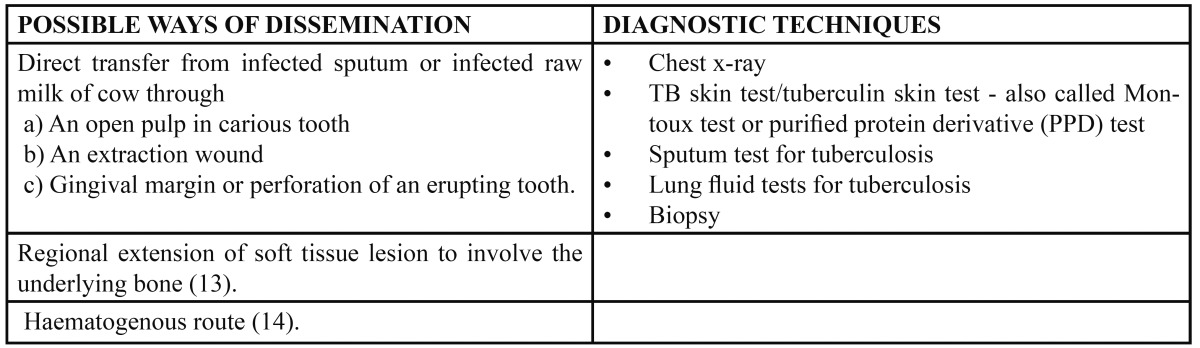
Possible ways of Dissemination and Diagnostic techniques of Tuberculosis infection.

Bacilli are possibly transferred from a primary focus in another part of the body and localized in the jaw, after trauma. Tuberculosis of the jaw causes slow necrosis of the bone and may involve the entire mandible. The destruction of the bone in radiographs appears as blurring of trabecular details with irregular areas of radiolucency. There is erosion of the cortex with little tendency to repair. Gradually the bone is replaced by soft tuberculous granulation tissue. Caseation appears at places followed by softening and liquefaction. A subperiosteal abscess then forms presenting as a painless, soft swelling. This cold abscess may burst either intra or extra orally forming single or multiple sinuses. Pathological fracture of mandible and sequestration may also occur. Usually tuberculosis of the mandible presents as multifocal lesion elsewhere in body, involving other bones and lungs.

Various investigation procedures are used for the diagnosis of tuberculosis are given in the  Table 1. Sometimes chest X-ray and Montoux test is non-contributory and gives false negative results. The patient may have been infected with M. tuberculosis, but doesn’t necessarily have an active disease or a patient has reduced immunity. These cases may give false positive results in Montoux test. For the reported cases all investigative procedures were carried out as per patient affordability and requirements reaching a definite diagnosis. CBCT was done to explore the third dimension and know the extent of lesion. Osteomyelitis and its associated changes in bone could be related to the blood supply and density of bone. Mandible is a dense bone with a single blood supply through inferior alveolar vessels. Thus when the dense bone is infected, the blood supply to the same is decreased which creates a favourable environment for the bacteria to grow. Early diagnosis of the lesion can reverse the changes that have occurred in the bone.

## Conclusion

Tuberculosis presents different clinical picture as per individual immunity and response towards infection. Although of rare occurrence, the differential diagnosis of tubercular osteomyelitis must always be kept in mind by clinicians, when routine therapy fails to bring about an improvement in the lesions of mandible. Since the involvement of bone occurs in late stages of the disease, the prognosis is poor and death from involvement of internal organs or from tubercular meningitis is common. However, if the lesion is primary and detected early, the disease is completely curable and can lead to reversal of all destructive bony changes.
